# The Hospital Library and Charities Bureau

**Published:** 1906-02-10

**Authors:** 


### THE HOSPITAL LIBRARY AND CHARITIES BUREAU.

This institution has been founded as a centre of reference in all matters concerning the establishment and management of charities. It is open to all interested in the subject on payment of a small annual fee, and inquiries by members can be made by post. Write for prospectus to the Librarian, The Hospital Buildings, 28 & 29 Southampton Street, Strand.

Inquiries of the Librarian are answered in this column free of charge. Inquiries to be answered promptly by post must be accompanied by a fee of 2s. 6d.

The sixty-seventh annual general meeting of the Newsvendors' Benevolent and Provident Institution will be held at the Institute of Printers, Tudor Street, E.C., on February 20, at seven o'clock, when Lord Glenesk will preside. The Mayor of Darlington and the editor of the *Yorkshire Post* have also promised to attend and take part in the proceedings.

